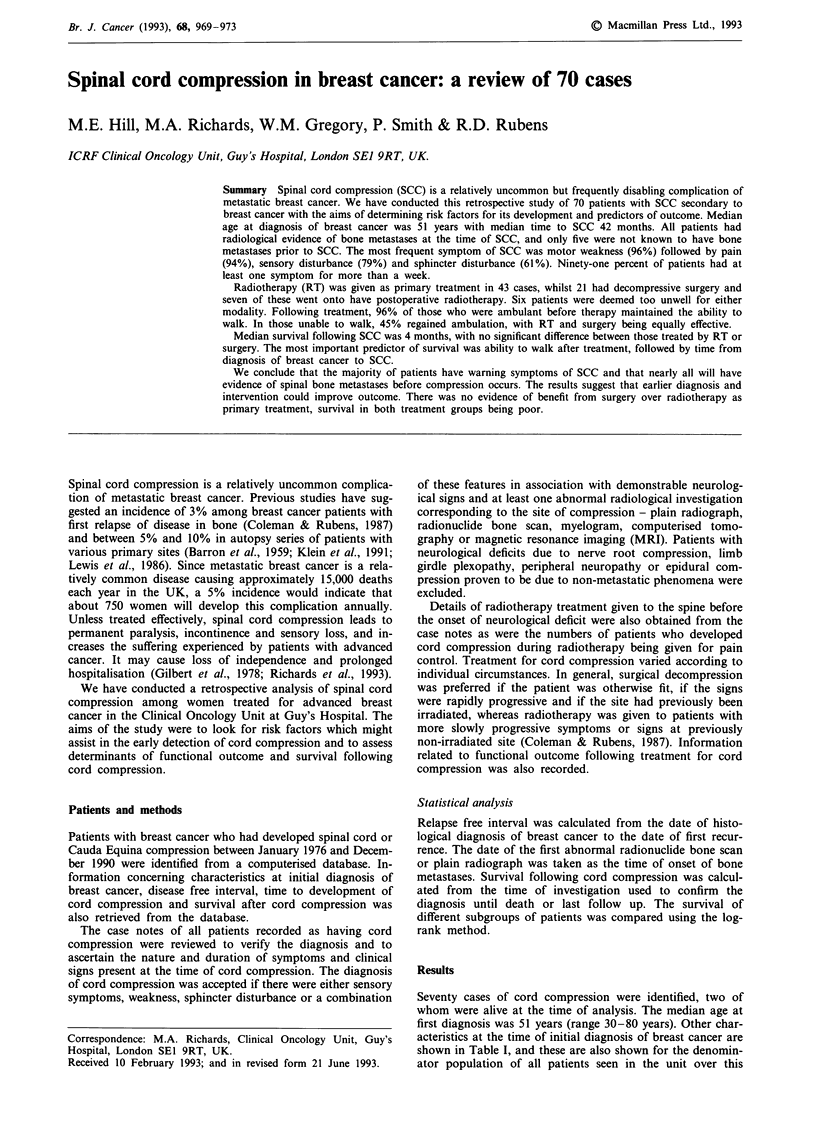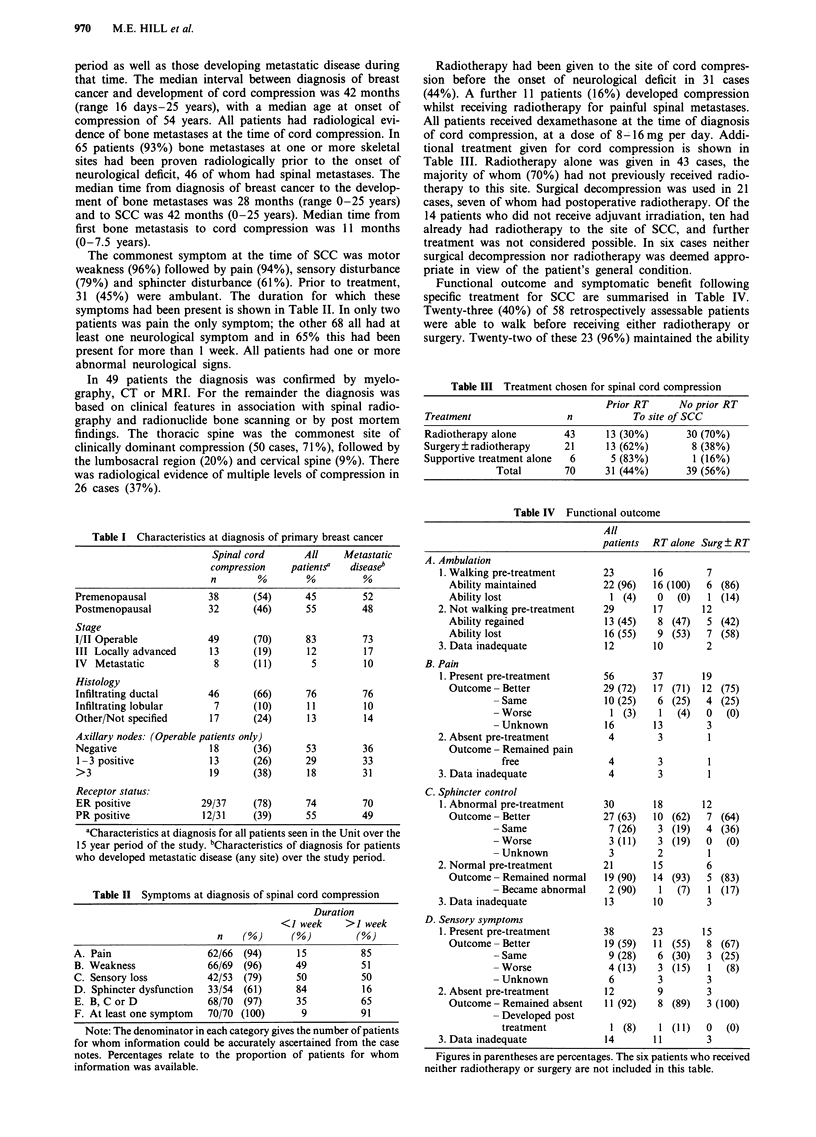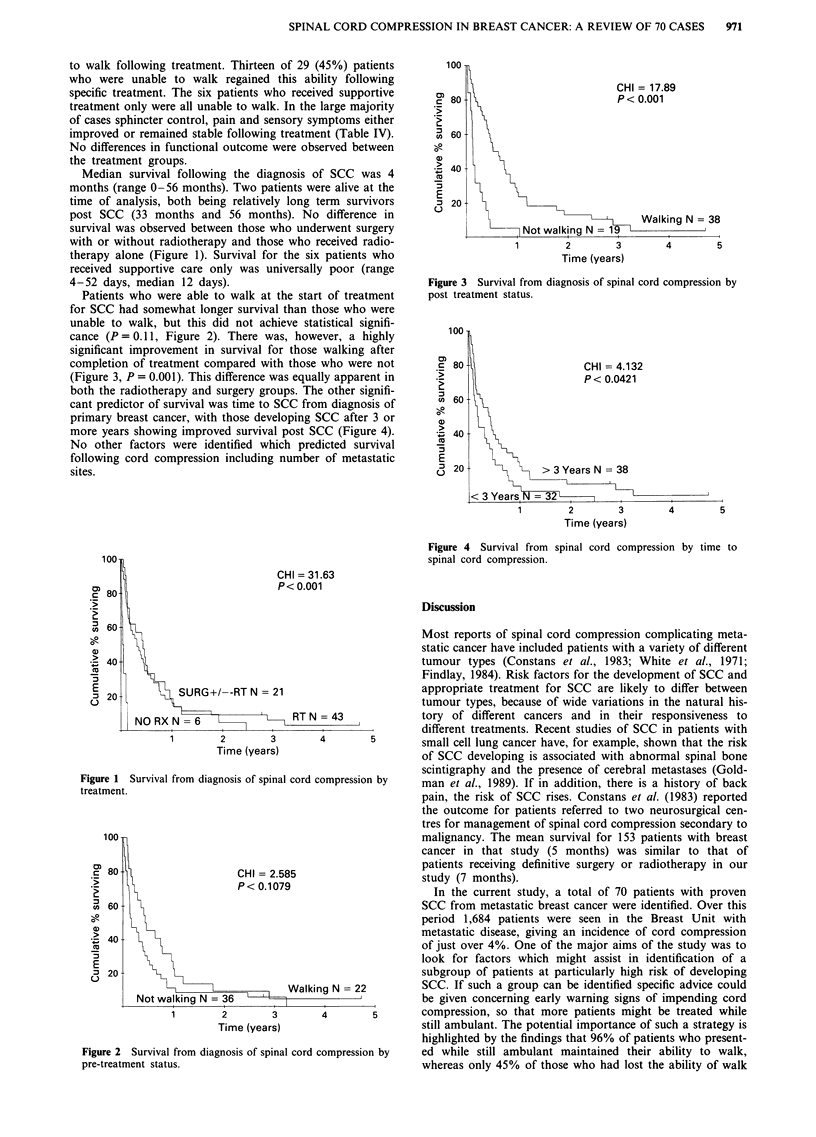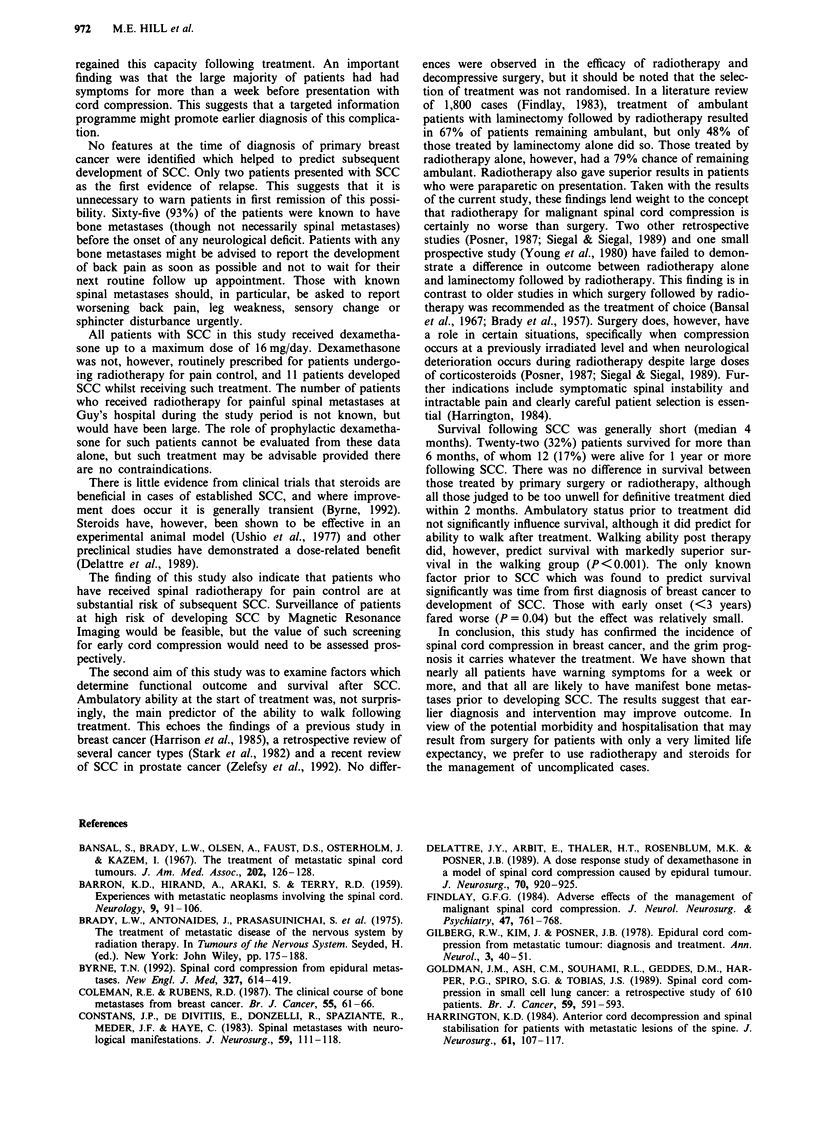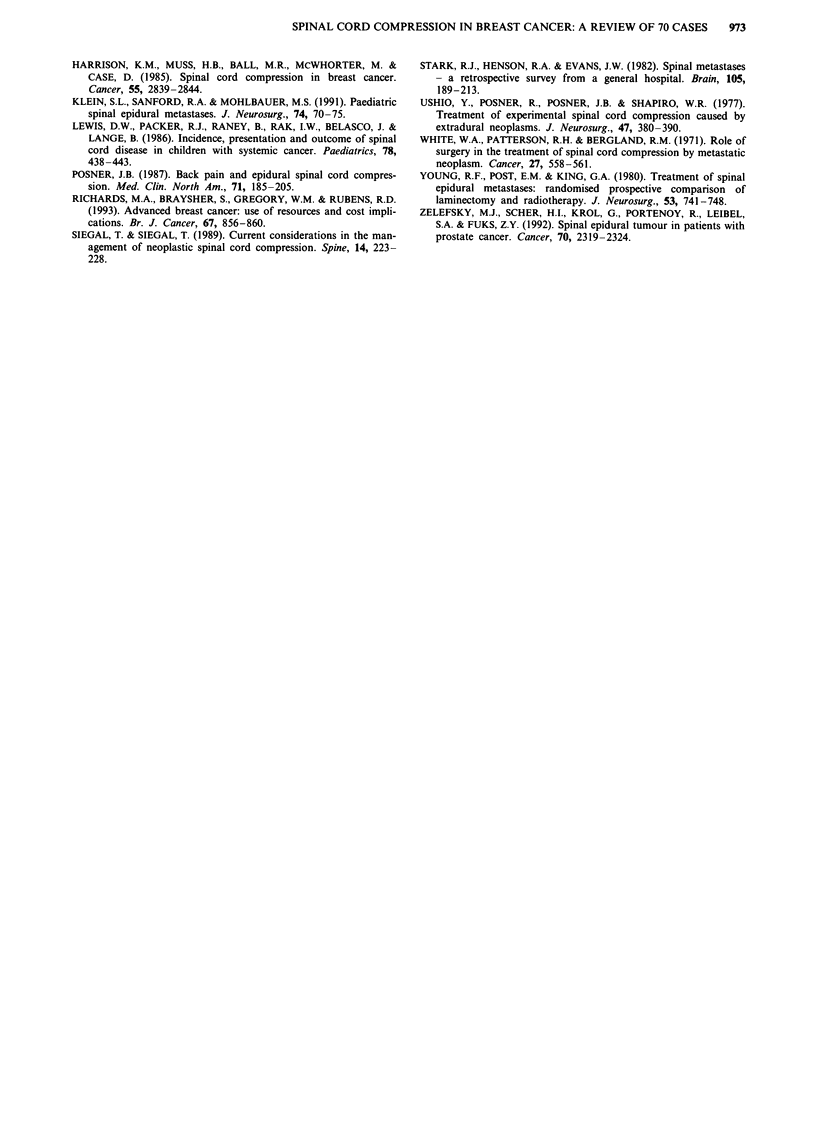# Spinal cord compression in breast cancer: a review of 70 cases.

**DOI:** 10.1038/bjc.1993.463

**Published:** 1993-11

**Authors:** M. E. Hill, M. A. Richards, W. M. Gregory, P. Smith, R. D. Rubens

**Affiliations:** ICRF Clinical Oncology Unit, Guy's Hospital, London, UK.

## Abstract

Spinal cord compression (SCC) is a relatively uncommon but frequently disabling complication of metastatic breast cancer. We have conducted this retrospective study of 70 patients with SCC secondary to breast cancer with the aims of determining risk factors for its development and predictors of outcome. Median age at diagnosis of breast cancer was 51 years with median time to SCC 42 months. All patients had radiological evidence of bone metastases at the time of SCC, and only five were not known to have bone metastases prior to SCC. The most frequent symptom of SCC was motor weakness (96%) followed by pain (94%), sensory disturbance (79%) and sphincter disturbance (61%). Ninety-one percent of patients had at least one symptom for more than a week. Radiotherapy (RT) was given as primary treatment in 43 cases, whilst 21 had decompressive surgery and seven of these went onto have postoperative radiotherapy. Six patients were deemed too unwell for either modality. Following treatment, 96% of those who were ambulant before therapy maintained the ability to walk. In those unable to walk, 45% regained ambulation, with RT and surgery being equally effective. Median survival following SCC was 4 months, with no significant difference between those treated by RT or surgery. The most important predictor of survival was ability to walk after treatment, followed by time from diagnosis of breast cancer to SCC. We conclude that the majority of patients have warning symptoms of SCC and that nearly all will have evidence of spinal bone metastases before compression occurs. The results suggest that earlier diagnosis and intervention could improve outcome. There was no evidence of benefit from surgery over radiotherapy as primary treatment, survival in both treatment groups being poor.


Br. J. Cancer (1993), 68, 969-973 © Macmillan Press Ltd., 1993

### Spinal cord compression in breast cancer: a review of 70 cases

M.E. Hill, M.A. Richards, W.M. Gregory, P. Smith & R.D. Rubens

*ICRF Clinical Oncology Unit, Guy's Hospital, London SE1 9RT, UK.*

**Summary** Spinal cord compression (SCC) is a relatively uncommon but frequently disabling complication of metastatic breast cancer. We have conducted this retrospective study of 70 patients with SCC secondary to breast cancer with the aims of determining risk factors for its development and predictors of outcome. Median age at diagnosis of breast cancer was 51 years with median time to SCC 42 months. All patients had radiological evidence of bone metastases at the time of SCC, and only five were not known to have bone metastases prior to SCC. The most frequent symptom of SCC was motor weakness (96%) followed by pain (94%), sensory disturbance (79%) and sphincter disturbance (61%). Ninety-one percent of patients had at least one symptom for more than a week.

Radiotherapy (RT) was given as primary treatment in 43 cases, whilst 21 had decompressive surgery and seven of these went onto have postoperative radiotherapy. Six patients were deemed too unwell for either modality. Following treatment, 96% of those who were ambulant before therapy maintained the ability to walk. In those unable to walk, 45% regained ambulation, with RT and surgery being equally effective.

Median survival following SCC was 4 months, with no significant difference between those treated by RT or surgery. The most important predictor of survival was ability to walk after treatment, followed by time from diagnosis of breast cancer to SCC.

We conclude that the majority of patients have warning symptoms of SCC and that nearly all will have evidence of spinal bone metastases before compression occurs. The results suggest that earlier diagnosis and intervention could improve outcome. There was no evidence of benefit from surgery over radiotherapy as primary treatment, survival in both treatment groups being poor.

Spinal cord compression is a relatively uncommon complication of metastatic breast cancer. Previous studies have suggested an incidence of 3% among breast cancer patients with first relapse of disease in bone (Coleman & Rubens, 1987) and between 5% and 10% in autopsy series of patients with various primary sites (Barron *et al.*, 1959; Klein *et al.*, 1991; Lewis *et al.*, 1986). Since metastatic breast cancer is a relatively common disease causing approximately 15,000 deaths each year in the UK, a 5% incidence would indicate that about 750 women will develop this complication annually. Unless treated effectively, spinal cord compression leads to permanent paralysis, incontinence and sensory loss, and increases the suffering experienced by patients with advanced cancer. It may cause loss of independence and prolonged hospitalisation (Gilbert *et al.*, 1978; Richards *et al.*, 1993).

We have conducted a retrospective analysis of spinal cord compression among women treated for advanced breast cancer in the Clinical Oncology Unit at Guy's Hospital. The aims of the study were to look for risk factors which might assist in the early detection of cord compression and to assess determinants of functional outcome and survival following cord compression.

### Patients and methods

Patients with breast cancer who had developed spinal cord or Cauda Equina compression between January 1976 and December 1990 were identified from a computerised database. Information concerning characteristics at initial diagnosis of breast cancer, disease free interval, time to development of cord compression and survival after cord compression was also retrieved from the database.

The case notes of all patients recorded as having cord compression were reviewed to verify the diagnosis and to ascertain the nature and duration of symptoms and clinical signs present at the time of cord compression. The diagnosis of cord compression was accepted if there were either sensory symptoms, weakness, sphincter disturbance or a combination of these features in association with demonstrable neurological signs and at least one abnormal radiological investigation corresponding to the site of compression – plain radiograph, radionuclide bone scan, myelogram, computerised tomography or magnetic resonance imaging (MRI). Patients with neurological deficits due to nerve root compression, limb girdle plexopathy, peripheral neuropathy or epidural compression proven to be due to non-metastatic phenomena were excluded.

Details of radiotherapy treatment given to the spine before the onset of neurological deficit were also obtained from the case notes as were the numbers of patients who developed cord compression during radiotherapy being given for pain control. Treatment for cord compression varied according to individual circumstances. In general, surgical decompression was preferred if the patient was otherwise fit, if the signs were rapidly progressive and if the site had previously been irradiated, whereas radiotherapy was given to patients with more slowly progressive symptoms or signs at previously non-irradiated site (Coleman & Rubens, 1987). Information related to functional outcome following treatment for cord compression was also recorded.

### *Statistical analysis*

Relapse free interval was calculated from the date of histological diagnosis of breast cancer to the date of first recurrence. The date of the first abnormal radionuclide bone scan or plain radiograph was taken as the time of onset of bone metastases. Survival following cord compression was calculated from the time of investigation used to confirm the diagnosis until death or last follow up. The survival of different subgroups of patients was compared using the log-rank method.

### Results

Seventy cases of cord compression were identified, two of whom were alive at the time of analysis. The median age at first diagnosis was 51 years (range 30–80 years). Other characteristics at the time of initial diagnosis of breast cancer are shown in Table I, and these are also shown for the denominator population of all patients seen in the unit over this

Correspondence: M.A. Richards, Clinical Oncology Unit, Guy's Hospital, London SE1 9RT, UK.
Received 10 February 1993; and in revised form 21 June 1993.

period as well as those developing metastatic disease during that time. The median interval between diagnosis of breast cancer and development of cord compression was 42 months (range 16 days–25 years), with a median age at onset of compression of 54 years. All patients had radiological evidence of bone metastases at the time of cord compression. In 65 patients (93%) bone metastases at one or more skeletal sites had been proven radiologically prior to the onset of neurological deficit, 46 of whom had spinal metastases. The median time from diagnosis of breast cancer to the development of bone metastases was 28 months (range 0–25 years) and to SCC was 42 months (0–25 years). Median time from first bone metastasis to cord compression was 11 months (0–7.5 years).

The commonest symptom at the time of SCC was motor weakness (96%) followed by pain (94%), sensory disturbance (79%) and sphincter disturbance (61%). Prior to treatment, 31 (45%) were ambulant. The duration for which these symptoms had been present is shown in Table II. In only two patients was pain the only symptom; the other 68 all had at least one neurological symptom and in 65% this had been present for more than 1 week. All patients had one or more abnormal neurological signs.

In 49 patients the diagnosis was confirmed by myelography, CT or MRI. For the remainder the diagnosis was based on clinical features in association with spinal radiography and radionuclide bone scanning or by post mortem findings. The thoracic spine was the commonest site of clinically dominant compression (50 cases, 71%), followed by the lumbosacral region (20%) and cervical spine (9%). There was radiological evidence of multiple levels of compression in 26 cases (37%).

**Table I** Characteristics at diagnosis of primary breast cancer

| | Spinal cord compression *n* | *%* | All patients[a] *%* | Metastatic disease[b] *%* |
|---|---|---|---|---|
| Premenopausal | 38 | (54) | 45 | 52 |
| Postmenopausal | 32 | (46) | 55 | 48 |
| *Stage* | | | | |
| I/II Operable | 49 | (70) | 83 | 73 |
| III Locally advanced | 13 | (19) | 12 | 17 |
| IV Metastatic | 8 | (11) | 5 | 10 |
| *Histology* | | | | |
| Infiltrating ductal | 46 | (66) | 76 | 76 |
| Infiltrating lobular | 7 | (10) | 11 | 10 |
| Other/Not specified | 17 | (24) | 13 | 14 |
| *Axillary nodes: (Operable patients only)* | | | | |
| Negative | 18 | (36) | 53 | 36 |
| 1–3 positive | 13 | (26) | 29 | 33 |
| >3 | 19 | (38) | 18 | 31 |
| *Receptor status:* | | | | |
| ER positive | 29/37 | (78) | 74 | 70 |
| PR positive | 12/31 | (39) | 55 | 49 |

[a]Characteristics at diagnosis for all patients seen in the Unit over the 15 year period of the study. [b]Characteristics of diagnosis for patients who developed metastatic disease (any site) over the study period.

**Table II** Symptoms at diagnosis of spinal cord compression

| | *n* | *(%)* | Duration <1 week *(%)* | Duration >1 week *(%)* |
|---|---|---|---|---|
| A. Pain | 62/66 | (94) | 15 | 85 |
| B. Weakness | 66/69 | (96) | 49 | 51 |
| C. Sensory loss | 42/53 | (79) | 50 | 50 |
| D. Sphincter dysfunction | 33/54 | (61) | 84 | 16 |
| E. B, C or D | 68/70 | (97) | 35 | 65 |
| F. At least one symptom | 70/70 | (100) | 9 | 91 |

Note: The denominator in each category gives the number of patients for whom information could be accurately ascertained from the case notes. Percentages relate to the proportion of patients for whom information was available.

Radiotherapy had been given to the site of cord compression before the onset of neurological deficit in 31 cases (44%). A further 11 patients (16%) developed compression whilst receiving radiotherapy for painful spinal metastases. All patients received dexamethasone at the time of diagnosis of cord compression, at a dose of 8–16 mg per day. Additional treatment given for cord compression is shown in Table III. Radiotherapy alone was given in 43 cases, the majority of whom (70%) had not previously received radiotherapy to this site. Surgical decompression was used in 21 cases, seven of whom had postoperative radiotherapy. Of the 14 patients who did not receive adjuvant irradiation, ten had already had radiotherapy to the site of SCC, and further treatment was not considered possible. In six cases neither surgical decompression nor radiotherapy was deemed appropriate in view of the patient's general condition.

Functional outcome and symptomatic benefit following specific treatment for SCC are summarised in Table IV. Twenty-three (40%) of 58 retrospectively assessable patients were able to walk before receiving either radiotherapy or surgery. Twenty-two of these 23 (96%) maintained the ability

**Table III** Treatment chosen for spinal cord compression

| *Treatment* | *n* | *Prior RT* To site of SCC | *No prior RT* To site of SCC |
|---|---|---|---|
| Radiotherapy alone | 43 | 13 (30%) | 30 (70%) |
| Surgery ± radiotherapy | 21 | 13 (62%) | 8 (38%) |
| Supportive treatment alone | 6 | 5 (83%) | 1 (16%) |
| Total | 70 | 31 (44%) | 39 (56%) |

**Table IV** Functional outcome

| | *All patients* | *RT alone* | *Surg ± RT* |
|---|---|---|---|
| *A. Ambulation* | | | |
| 1. Walking pre-treatment | 23 | 16 | 7 |
| Ability maintained | 22 (96) | 16 (100) | 6 (86) |
| Ability lost | 1 (4) | 0 (0) | 1 (14) |
| 2. Not walking pre-treatment | 29 | 17 | 12 |
| Ability regained | 13 (45) | 8 (47) | 5 (42) |
| Ability lost | 16 (55) | 9 (53) | 7 (58) |
| 3. Data inadequate | 12 | 10 | 2 |
| *B. Pain* | | | |
| 1. Present pre-treatment | 56 | 37 | 19 |
| Outcome – Better | 29 (72) | 17 (71) | 12 (75) |
| – Same | 10 (25) | 6 (25) | 4 (25) |
| – Worse | 1 (3) | 1 (4) | 0 (0) |
| – Unknown | 16 | 13 | 3 |
| 2. Absent pre-treatment | 4 | 3 | 1 |
| Outcome – Remained pain free | 4 | 3 | 1 |
| 3. Data inadequate | 4 | 3 | 1 |
| *C. Sphincter control* | | | |
| 1. Abnormal pre-treatment | 30 | 18 | 12 |
| Outcome – Better | 27 (63) | 10 (62) | 7 (64) |
| – Same | 7 (26) | 3 (19) | 4 (36) |
| – Worse | 3 (11) | 3 (19) | 0 (0) |
| – Unknown | 3 | 2 | 1 |
| 2. Normal pre-treatment | 21 | 15 | 6 |
| Outcome – Remained normal | 19 (90) | 14 (93) | 5 (83) |
| – Became abnormal | 2 (90) | 1 (7) | 1 (17) |
| 3. Data inadequate | 13 | 10 | 3 |
| *D. Sensory symptoms* | | | |
| 1. Present pre-treatment | 38 | 23 | 15 |
| Outcome – Better | 19 (59) | 11 (55) | 8 (67) |
| – Same | 9 (28) | 6 (30) | 3 (25) |
| – Worse | 4 (13) | 3 (15) | 1 (8) |
| – Unknown | 6 | 3 | 3 |
| 2. Absent pre-treatment | 12 | 9 | 3 |
| Outcome – Remained absent | 11 (92) | 8 (89) | 3 (100) |
| – Developed post treatment | 1 (8) | 1 (11) | 0 (0) |
| 3. Data inadequate | 14 | 11 | 3 |

Figures in parentheses are percentages. The six patients who received neither radiotherapy or surgery are not included in this table.

to walk following treatment. Thirteen of 29 (45%) patients who were unable to walk regained this ability following specific treatment. The six patients who received supportive treatment only were all unable to walk. In the large majority of cases sphincter control, pain and sensory symptoms either improved or remained stable following treatment (Table IV). No differences in functional outcome were observed between the treatment groups.

Median survival following the diagnosis of SCC was 4 months (range 0–56 months). Two patients were alive at the time of analysis, both being relatively long term survivors post SCC (33 months and 56 months). No difference in survival was observed between those who underwent surgery with or without radiotherapy and those who received radiotherapy alone (Figure 1). Survival for the six patients who received supportive care only was universally poor (range 4–52 days, median 12 days).

Patients who were able to walk at the start of treatment for SCC had somewhat longer survival than those who were unable to walk, but this did not achieve statistical significance ($P = 0.11$, Figure 2). There was, however, a highly significant improvement in survival for those walking after completion of treatment compared with those who were not (Figure 3, $P = 0.001$). This difference was equally apparent in both the radiotherapy and surgery groups. The other significant predictor of survival was time to SCC from diagnosis of primary breast cancer, with those developing SCC after 3 or more years showing improved survival post SCC (Figure 4). No other factors were identified which predicted survival following cord compression including number of metastatic sites.

**Figure 1** Survival from diagnosis of spinal cord compression by treatment.

**Figure 2** Survival from diagnosis of spinal cord compression by pre-treatment status.

**Figure 3** Survival from diagnosis of spinal cord compression by post treatment status.

**Figure 4** Survival from spinal cord compression by time to spinal cord compression.

### Discussion

Most reports of spinal cord compression complicating metastatic cancer have included patients with a variety of different tumour types (Constans *et al.*, 1983; White *et al.*, 1971; Findlay, 1984). Risk factors for the development of SCC and appropriate treatment for SCC are likely to differ between tumour types, because of wide variations in the natural history of different cancers and in their responsiveness to different treatments. Recent studies of SCC in patients with small cell lung cancer have, for example, shown that the risk of SCC developing is associated with abnormal spinal bone scintigraphy and the presence of cerebral metastases (Goldman *et al.*, 1989). If in addition, there is a history of back pain, the risk of SCC rises. Constans *et al.* (1983) reported the outcome for patients referred to two neurosurgical centres for management of spinal cord compression secondary to malignancy. The mean survival for 153 patients with breast cancer in that study (5 months) was similar to that of patients receiving definitive surgery or radiotherapy in our study (7 months).

In the current study, a total of 70 patients with proven SCC from metastatic breast cancer were identified. Over this period 1,684 patients were seen in the Breast Unit with metastatic disease, giving an incidence of cord compression of just over 4%. One of the major aims of the study was to look for factors which might assist in identification of a subgroup of patients at particularly high risk of developing SCC. If such a group can be identified specific advice could be given concerning early warning signs of impending cord compression, so that more patients might be treated while still ambulant. The potential importance of such a strategy is highlighted by the findings that 96% of patients who presented while still ambulant maintained their ability to walk, whereas only 45% of those who had lost the ability of walk regained this capacity following treatment. An important finding was that the large majority of patients had had symptoms for more than a week before presentation with cord compression. This suggests that a targeted information programme might promote earlier diagnosis of this complication.

No features at the time of diagnosis of primary breast cancer were identified which helped to predict subsequent development of SCC. Only two patients presented with SCC as the first evidence of relapse. This suggests that it is unnecessary to warn patients in first remission of this possibility. Sixty-five (93%) of the patients were known to have bone metastases (though not necessarily spinal metastases) before the onset of any neurological deficit. Patients with any bone metastases might be advised to report the development of back pain as soon as possible and not to wait for their next routine follow up appointment. Those with known spinal metastases should, in particular, be asked to report worsening back pain, leg weakness, sensory change or sphincter disturbance urgently.

All patients with SCC in this study received dexamethasone up to a maximum dose of 16 mg/day. Dexamethasone was not, however, routinely prescribed for patients undergoing radiotherapy for pain control, and 11 patients developed SCC whilst receiving such treatment. The number of patients who received radiotherapy for painful spinal metastases at Guy's hospital during the study period is not known, but would have been large. The role of prophylactic dexamethasone for such patients cannot be evaluated from these data alone, but such treatment may be advisable provided there are no contraindications.

There is little evidence from clinical trials that steroids are beneficial in cases of established SCC, and where improvement does occur it is generally transient (Byrne, 1992). Steroids have, however, been shown to be effective in an experimental animal model (Ushio *et al.*, 1977) and other preclinical studies have demonstrated a dose-related benefit (Delattre *et al.*, 1989).

The finding of this study also indicate that patients who have received spinal radiotherapy for pain control are at substantial risk of subsequent SCC. Surveillance of patients at high risk of developing SCC by Magnetic Resonance Imaging would be feasible, but the value of such screening for early cord compression would need to be assessed prospectively.

The second aim of this study was to examine factors which determine functional outcome and survival after SCC. Ambulatory ability at the start of treatment was, not surprisingly, the main predictor of the ability to walk following treatment. This echoes the findings of a previous study in breast cancer (Harrison *et al.*, 1985), a retrospective review of several cancer types (Stark *et al.*, 1982) and a recent review of SCC in prostate cancer (Zelefsy *et al.*, 1992). No differences were observed in the efficacy of radiotherapy and decompressive surgery, but it should be noted that the selection of treatment was not randomised. In a literature review of 1,800 cases (Findlay, 1983), treatment of ambulant patients with laminectomy followed by radiotherapy resulted in 67% of patients remaining ambulant, but only 48% of those treated by laminectomy alone did so. Those treated by radiotherapy alone, however, had a 79% chance of remaining ambulant. Radiotherapy also gave superior results in patients who were paraparetic on presentation. Taken with the results of the current study, these findings lend weight to the concept that radiotherapy for malignant spinal cord compression is certainly no worse than surgery. Two other retrospective studies (Posner, 1987; Siegal & Siegal, 1989) and one small prospective study (Young *et al.*, 1980) have failed to demonstrate a difference in outcome between radiotherapy alone and laminectomy followed by radiotherapy. This finding is in contrast to older studies in which surgery followed by radiotherapy was recommended as the treatment of choice (Bansal *et al.*, 1967; Brady *et al.*, 1957). Surgery does, however, have a role in certain situations, specifically when compression occurs at a previously irradiated level and when neurological deterioration occurs during radiotherapy despite large doses of corticosteroids (Posner, 1987; Siegal & Siegal, 1989). Further indications include symptomatic spinal instability and intractable pain and clearly careful patient selection is essential (Harrington, 1984).

Survival following SCC was generally short (median 4 months). Twenty-two (32%) patients survived for more than 6 months, of whom 12 (17%) were alive for 1 year or more following SCC. There was no difference in survival between those treated by primary surgery or radiotherapy, although all those judged to be too unwell for definitive treatment died within 2 months. Ambulatory status prior to treatment did not significantly influence survival, although it did predict for ability to walk after treatment. Walking ability post therapy did, however, predict survival with markedly superior survival in the walking group ($P < 0.001$). The only known factor prior to SCC which was found to predict survival significantly was time from first diagnosis of breast cancer to development of SCC. Those with early onset (<3 years) fared worse ($P = 0.04$) but the effect was relatively small.

In conclusion, this study has confirmed the incidence of spinal cord compression in breast cancer, and the grim prognosis it carries whatever the treatment. We have shown that nearly all patients have warning symptoms for a week or more, and that all are likely to have manifest bone metastases prior to developing SCC. The results suggest that earlier diagnosis and intervention may improve outcome. In view of the potential morbidity and hospitalisation that may result from surgery for patients with only a very limited life expectancy, we prefer to use radiotherapy and steroids for the management of uncomplicated cases.

### References

BANSAL, S., BRADY, L.W., OLSEN, A., FAUST, D.S., OSTERHOLM, J. & KAZEM, I. (1967). The treatment of metastatic spinal cord tumours. *J. Am. Med. Assoc.*, **202**, 126–128.

BARRON, K.D., HIRAND, A., ARAKI, S. & TERRY, R.D. (1959). Experiences with metastatic neoplasms involving the spinal cord. *Neurology*, **9**, 91–106.

BRADY, L.W., ANTONAIDES, J., PRASASUINICHAI, S. *et al.* (1975). The treatment of metastatic disease of the nervous system by radiation therapy. In *Tumours of the Nervous System*. Seyded, H. (ed.). New York: John Wiley, pp. 175–188.

BYRNE, T.N. (1992). Spinal cord compression from epidural metastases. *New Engl. J. Med*, **327**, 614–419.

COLEMAN, R.E. & RUBENS, R.D. (1987). The clinical course of bone metastases from breast cancer. *Br. J. Cancer*, **55**, 61–66.

CONSTANS, J.P., DE DIVITIIS, E., DONZELLI, R., SPAZIANTE, R., MEDER, J.F. & HAYE, C. (1983). Spinal metastases with neurological manifestations. *J. Neurosurg.*, **59**, 111–118.

DELATTRE, J.Y., ARBIT, E., THALER, H.T., ROSENBLUM, M.K. & POSNER, J.B. (1989). A dose response study of dexamethasone in a model of spinal cord compression caused by epidural tumour. *J. Neurosurg.*, **70**, 920–925.

FINDLAY, G.F.G. (1984). Adverse effects of the management of malignant spinal cord compression. *J. Neurol. Neurosurg. & Psychiatry*, **47**, 761–768.

GILBERG, R.W., KIM, J. & POSNER, J.B. (1978). Epidural cord compression from metastatic tumour: diagnosis and treatment. *Ann. Neurol.*, **3**, 40–51.

GOLDMAN, J.M., ASH, C.M., SOUHAMI, R.L., GEDDES, D.M., HARPER, P.G., SPIRO, S.G. & TOBIAS, J.S. (1989). Spinal cord compression in small cell lung cancer: a retrospective study of 610 patients. *Br. J. Cancer*, **59**, 591–593.

HARRINGTON, K.D. (1984). Anterior cord decompression and spinal stabilisation for patients with metastatic lesions of the spine. *J. Neurosurg.*, **61**, 107–117.

HARRISON, K.M., MUSS, H.B., BALL, M.R., MCWHORTER, M. & CASE, D. (1985). Spinal cord compression in breast cancer. *Cancer*, **55**, 2839–2844.

KLEIN, S.L., SANFORD, R.A. & MOHLBAUER, M.S. (1991). Paediatric spinal epidural metastases. *J. Neurosurg.*, **74**, 70–75.

LEWIS, D.W., PACKER, R.J., RANEY, B., RAK, I.W., BELASCO, J. & LANGE, B. (1986). Incidence, presentation and outcome of spinal cord disease in children with systemic cancer. *Paediatrics*, **78**, 438–443.

POSNER, J.B. (1987). Back pain and epidural spinal cord compression. *Med. Clin. North Am.*, **71**, 185–205.

RICHARDS, M.A., BRAYSHER, S., GREGORY, W.M. & RUBENS, R.D. (1993). Advanced breast cancer: use of resources and cost implications. *Br. J. Cancer*, **67**, 856–860.

SIEGAL, T. & SIEGAL, T. (1989). Current considerations in the management of neoplastic spinal cord compression. *Spine*, **14**, 223–228.

STARK, R.J., HENSON, R.A. & EVANS, J.W. (1982). Spinal metastases – a retrospective survey from a general hospital. *Brain*, **105**, 189–213.

USHIO, Y., POSNER, R., POSNER, J.B. & SHAPIRO, W.R. (1977). Treatment of experimental spinal cord compression caused by extradural neoplasms. *J. Neurosurg.*, **47**, 380–390.

WHITE, W.A., PATTERSON, R.H. & BERGLAND, R.M. (1971). Role of surgery in the treatment of spinal cord compression by metastatic neoplasm. *Cancer*, **27**, 558–561.

YOUNG, R.F., POST, E.M. & KING, G.A. (1980). Treatment of spinal epidural metastases: randomised prospective comparison of laminectomy and radiotherapy. *J. Neurosurg.*, **53**, 741–748.

ZELEFSKY, M.J., SCHER, H.I., KROL, G., PORTENOY, R., LEIBEL, S.A. & FUKS, Z.Y. (1992). Spinal epidural tumour in patients with prostate cancer. *Cancer*, **70**, 2319–2324.